# Gut Contents as Direct Indicators for Trophic Relationships in the Cambrian Marine Ecosystem

**DOI:** 10.1371/journal.pone.0052200

**Published:** 2012-12-26

**Authors:** Jean Vannier

**Affiliations:** Laboratoire de géologie de Lyon: Terre, Planètes, Environnement, Université de Lyon, Université Lyon 1, Villeurbanne, France; Raymond M. Alf Museum of Paleontology, United States of America

## Abstract

Present-day ecosystems host a huge variety of organisms that interact and transfer mass and energy via a cascade of trophic levels. When and how this complex machinery was established remains largely unknown. Although exceptionally preserved biotas clearly show that Early Cambrian animals had already acquired functionalities that enabled them to exploit a wide range of food resources, there is scant direct evidence concerning their diet and exact trophic relationships. Here I describe the gut contents of *Ottoia prolifica*, an abundant priapulid worm from the middle Cambrian (Stage 5) Burgess Shale biota. I identify the undigested exoskeletal remains of a wide range of small invertebrates that lived at or near the water sediment interface such as hyolithids, brachiopods, different types of arthropods, polychaetes and wiwaxiids. This set of direct fossil evidence allows the first detailed reconstruction of the diet of a 505-million-year-old animal. *Ottoia* was a dietary generalist and had no strict feeding regime. It fed on both living individuals and decaying organic matter present in its habitat. The feeding behavior of *Ottoia* was remarkably simple, reduced to the transit of food through an eversible pharynx and a tubular gut with limited physical breakdown and no storage. The recognition of generalist feeding strategies, exemplified by *Ottoia*, reveals key-aspects of modern-style trophic complexity in the immediate aftermath of the Cambrian explosion. It also shows that the middle Cambrian ecosystem was already too complex to be understood in terms of simple linear dynamics and unique pathways.

## Introduction

The study of exceptionally preserved Cambrian biotas [e.g., Burgess Shale [1], [2], Chengjiang [3], [4], Sirius Passet [5]–[7] and Emu Bay Shale [8]–[10] has led to accurate reconstructions of the anatomy, lifestyles [11]–[13], visual properties [10], and even behaviors [14], [15] of early animals. However, information is lacking concerning their interactions within the food chain and their diet. The functioning of the Cambrian ecosystem has mainly been addressed through a combination of indirect fossil evidence supported by modern analogues [16]. Typically, the feeding types (e.g. predation vs. particle-feeding) and strategies (sediment-eating vs. carnivory) of most Cambrian animals have been inferred from the morphofunctional analysis of their food-gathering apparatuses/limbs [17]–[20] and digestive systems [21]. The predatory habit of anomalocaridids, for example, is supported by evidence from their frontal appendages, mouth apparatus [22]–[24] and sophisticated eyes [10], but there is no direct evidence of what organisms they actually preyed upon. Mechanical models using finite element analysis [25] and recent studies of the oral cone [26] contradict the view that anamolocaridids were durophagous predators able to perform strong biting motions and to inflict wounds on hard exoskeletons [24], [27], [28]. The contents from coprolites [29] provide a degree of trophic resolution but cannot be tied to particular predators although some coprolites composed entirely of crushed skeletal elements from the Cambrian of California, Utah, Canada (Burgess Shale) and Australia [30] may have been produced by arthropods with robust gnathobasic appendages such as *Sidneyia* [31]. Rare fossil associations [32] and trace fossils [33] have suggested possible hunting or scavenging behaviors but these relationships require quantification. Qualitative and quantitative analyses of the communities from the Burgess Shale [2], [34] and the Maotianshan Shale [35]–[37] have provided detailed information on the diversity of ecological types and the presumed organization of the early and middle Cambrian ecosystems but do not tell us about the exact trophic links between species. Recent theoretical models [38] have predicted strong similarities between the trophic organization of Cambrian food webs and modern ones but lack detailed testing by fossil evidences. By contrast, the analysis of gut contents presented here and exemplified by the priapulid worm *Ottoia prolifica* from the middle Cambrian Burgess Shale provides direct and detailed evidence for trophic relationships and new insights both into the actual diet and feeding behavior of Cambrian animals. The case of priapulids reveals the potential of a source of information that has long been considered as relatively limited and anecdotal [39], [40]. A noticeable exception though is S. Conway Morris’ comprehensive work [39] on the priapulid worms from the Burgess Shale in which the gut contents of *Ottoia* are first described. This pioneer work is important in that it led to the concept of *Ottoia* as an iconic Cambrian predator and formed the basis of my study. My results also invite reassessment of the function and the complexity of Cambrian marine food webs where animals, for the first time in their history, played a major role in the transfer of mass and energy. The interpretations here also challenge the notion of strict feeding regimes and linear food chain and provide support for a marine trophic web where energy flow circulated via multiple animal interactions and parallel pathways [41], as it does in present-day ecosystems.

## Materials and Methods

**Figure 1:**
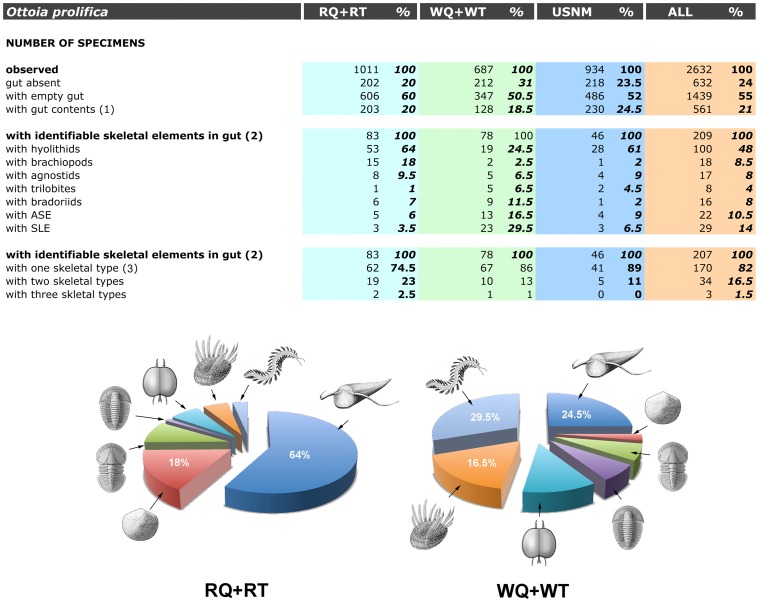
Count data and composition of the gut contents of *Ottoia prolifica*, from the middle Cambrian Burgess Shale Formation (Series 3, Stage 5; see [**45**]). The pie diagrams illustrate differences in the diet of *Ottoia* from the Raymond Quarry (RQ+RT) and the Walcott (WQ+WT) Quarry. Hyolithids dominate in the gut contents from the Raymond Quarry followed in decreasing order by brachiopods, agnostids, trilobites, bradoriids, ASE (presumed wiwaxiids), SLE (presumed polychaetes) and trilobites. In the Walcott Quarry, three almost equally represented groups (SLE, hyolithids and ASE) are prevalent, followed by bradoriids, trilobites, agnostids and brachiopods. (1) guts containing skeletal fragments and/or undetermined material and a variable proportion of sediment; (2) guts containing skeletal elements or fragments that belong to animal species present in the Burgess Shale biota; (3) guts containing elements that belong to a single species (e.g. only hyolithid skeletal elements). Empty guts generally appear as a colored or reflective strip running axially from the pharynx to the anus. ASE, almond-shape elements (presumed wiwaxiid sclerites); RQ, RT, collection specimens from the Raymond Quarry and talus (Royal Ontario Museum); SLE, setae-like elements (presumed polychaete chaetae); USNM, collection specimens from the National Museum of Natural History, Smithsonian Institution, Washington D.C.; WQ, WT, collection specimens from the Walcott Quarry and talus (Royal Ontario Museum). Raw data in Table S1.

Our fossil material comes from two stratigraphic horizons in the middle Cambrian Burgess Shale Member: 1) the Walcott Quarry Member, characterized by fossiliferous, finely laminated, calcareous, siltstones and silty graphitic mudstones, typically with a weathered horizontally-banded appearance; and 2) the slightly younger Raymond Quarry Member, characterized by grey, greenish and brown layered blocky-slaty mudstone [1], [2], [42]–[44]. The *Ottoia* specimens kept in the collections of the National Museum of Natural History, Smithsonian Institution, Washington D.C. (USNM), all come from excavations at Walcott’s original site (the so-called Phyllopod Bed within the Walcott Quarry Member). Those from the Royal Ontario Museum (ROM) collections, Toronto, were collected from both the Raymond and Walcott Quarry Members (RQ, RT and WQ, WT numbers respectively) in successive seasons of excavations and talus picking (RT, WT) between 1975 and 2000 by Royal Ontario Museum parties led by D. Collins. Altogether more than 2,600 specimens of *Ottoia prolifica* were examined, only a small percentage had preserved gut contents (Fig. 1, Table S1). The recent priapulid *Priapulus caudatus* was collected from the Gullmar fjord near the Sven Lovén Centre for Marine Sciences at Kristineberg, Sweden and from near The White Sea Biological Station “Kartesh” (WSBS), Russia. Digital photography (with polarizing filters to increase contrast of anatomical features), scanning electron microscopy and Energy-dispersive X-ray spectroscopy (EDX) analysis were used to study the morphology and chemical composition of the fossil and Recent material. The global chronostratigraphic subdivision of the Cambrian System is currently in the process of ratification by the International Union of Geological Sciences (IUGS). The Burgess Shale Formation belongs to Series 3, Stage 5 (see recent provisional chart [45]). For convenience, I maintain usage of “middle Cambrian” for this formation.

This research does not involve human participants. I obtained permission to study the Burgess Shale fossil collections from the Royal Ontario Museum (ROM,Toronto) and the Smithsonian National Museum of Natural History (USNM, Washington D.C.) from Jean-Bernard Caron and Douglas Erwin, respectively. The majority of specimens were studied in the ROM and the USNM. A small number of them were obtained on loan and returned.

## Results

### Gut Content Analysis

**Figure 2:**
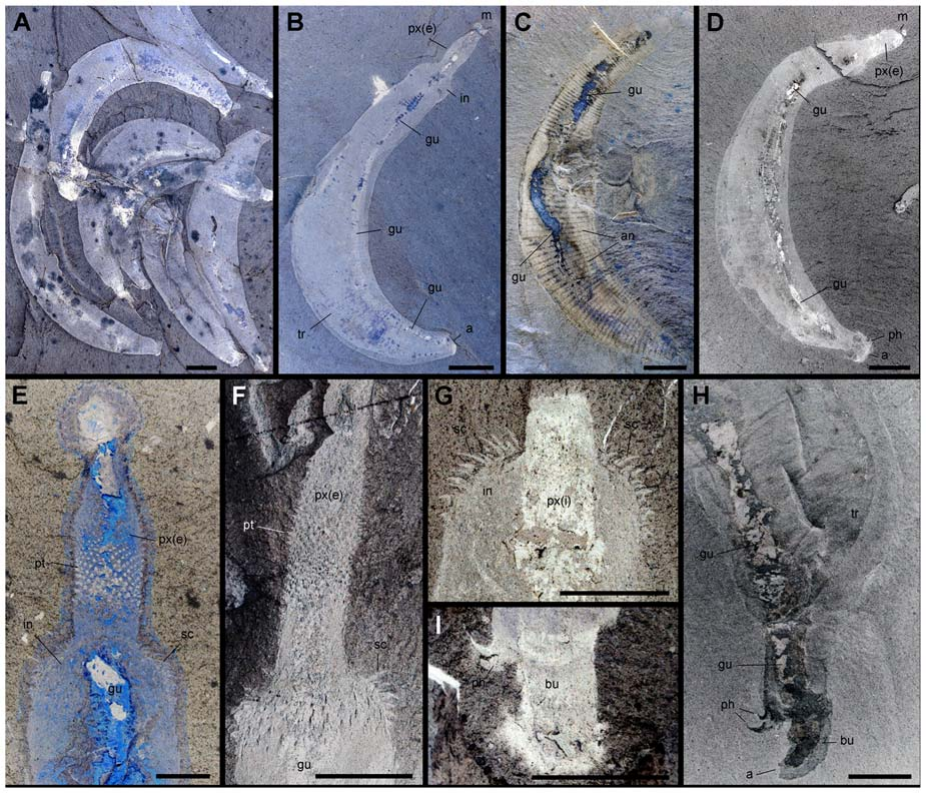
General morphology of *Ottoia prolifica* **from the middle Cambrian Burgess Shale.** A, ROM 61780a, high concentration of complete specimens. B–D, ROM 61759, ROM 61752 and ROM 61757, complete specimens. E, F, ROM 61751 and ROM 61765, details of anterior part. G, ROM 61760, details of introvert bearing curved scalids. H, I, ROM 61769 and ROM 61764, details of posterior part showing bursa and posterior hooks. Abbreviations: a, anus; an, trunk annulation; bu, bursa; gu, gut; in, introvert; m, mouth; ph, posterior hook; pt, pharyngeal teeth; px, pharynx; px(e), everted pharynx; px(i), inverted pharynx; sc, scalid; tr, trunk. Scale bar: 1 cm for A–D and 5 mm for E–I.

**Figure 3:**
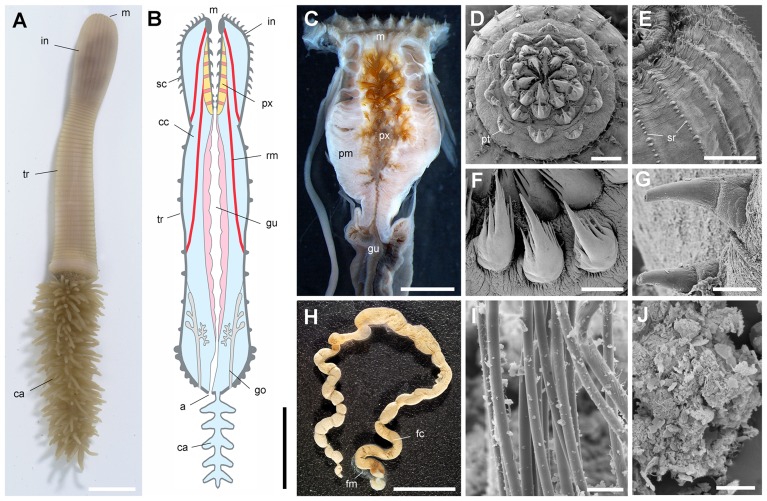
General morphology of Recent priapulid worms exemplified by *Priapulus caudatus* collected from near the Kristineberg Marine Station, Gullmarfjord, Sweden, depth ca. 30 m. A, B, general view of a live specimen in sea water and simplified section through body showing major anatomical features. C, section through pharynx (sclerotized pharyngeal teeth in orange; introvert removed. D, F, frontal view of a slightly everted pharynx showing pentagonal pattern of pharyngeal teeth around mouth opening and details of pharyngeal teeth. *E, G,* scalid rows along bulbous introvert and details of scalids (tip perforated). H–J, feces of *Priapulus caudatus* filled with compacted undigested material and enclosed by a transparent membrane, bundles of undigested polychaete chaetae and undetermined gut contents (mainly sediment and detritus of various origin). D–G, I, J, are scanning electron micrographs of dessicated specimens. Abbreviations: a, anus; an, trunk annulation; bu, bursa; ca, caudal appendage; cc, coelomic cavity; fc, feces contents; fm, feces membrane; go, gonads; gu, gut; in, introvert; m, mouth; pm, pharyngeal muscles; pt, pharyngeal tooth; px, pharynx; rm, retractor muscle; sc, scalid; sr, scalid row; tr, trunk. Scale bar: 1 cm for A, B; 5 mm for C; 500 µm for D, E, H; 200 µm for F; 100 µm for G; 10 µm for I; 5 µm for J.

**Figure 4:**
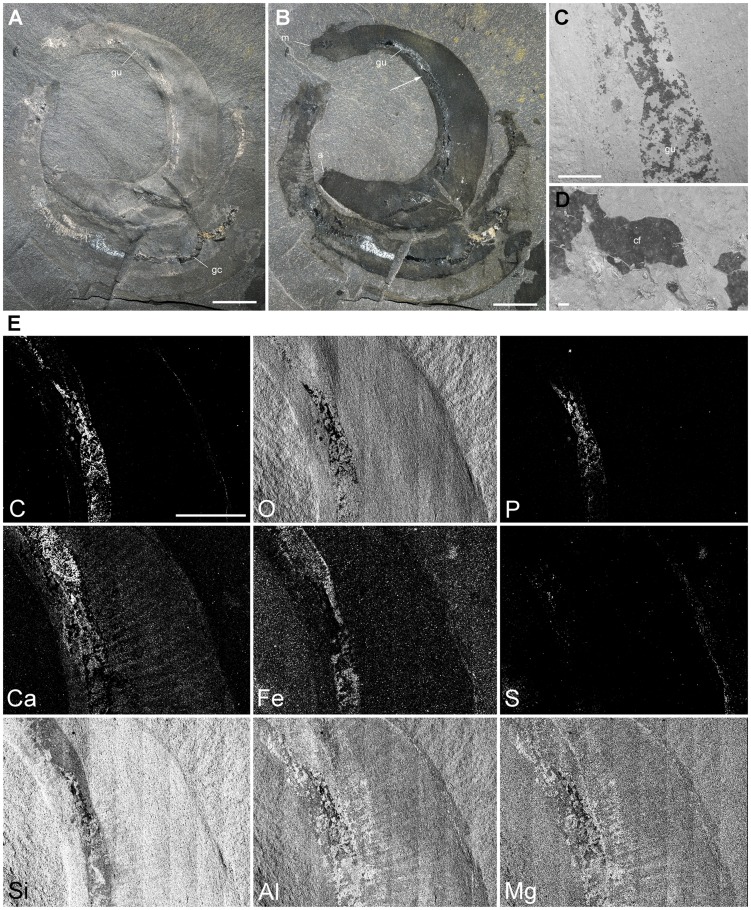
Elemental mapping of the gut of *Ottoia prolifica* **from the middle Cambrian Burgess Shale.** The mapping reveals anatomical partitioning of the gut, with elevated C, Fe and P that probably reflects its organic-rich original composition and early diagenetic mineralizations in pyrite, apatite or calcite. A–E, ROM 61758b. A, B, general view under normal and polarized light (white arrow to indicate mapped area). C, D, back scattered image of gut showing patches of carbonaceous film; this film is interpreted as remains of the gut wall, rather than gut contents. E, elemental mapping. Abbreviations: a, anus; cf, carbonaceous film; gc, gut content; gu, gut; m, mouth. Scale bar: 1 cm for A, B; 5 mm for E; 1 mm for C; 20 µm for D.

**Figure 5:**
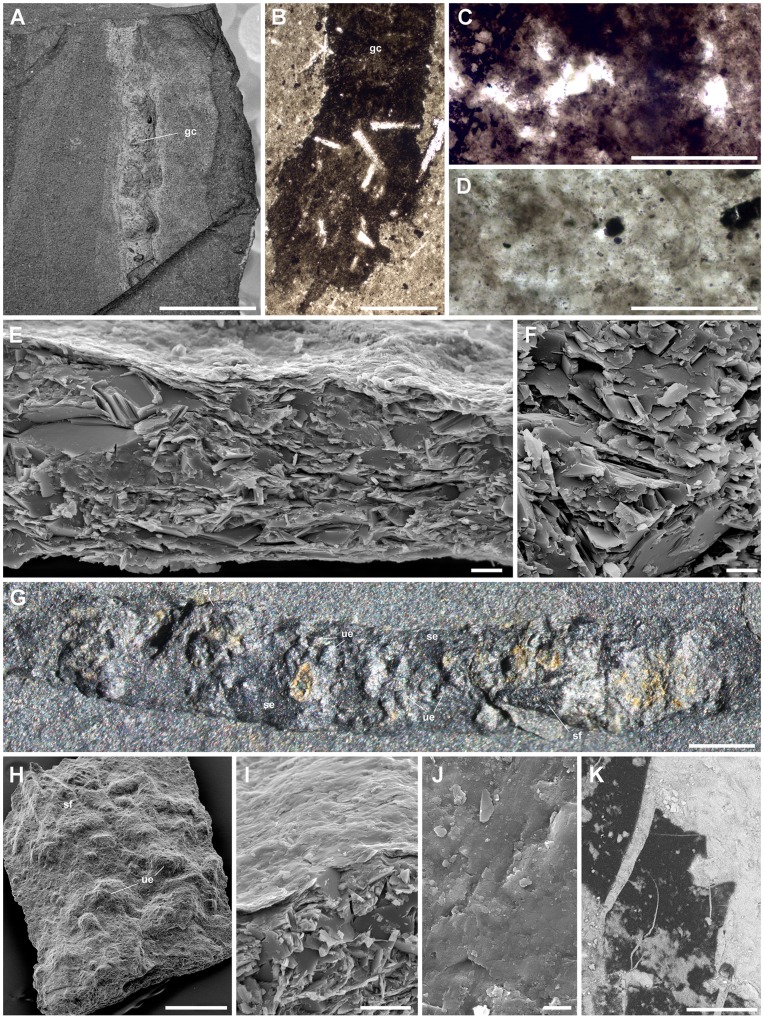
Sedimentary ingesta within the gut of *Ottoia prolifica* **from the middle Cambrian Burgess Shale Formation.** A–D, USNM 196195, three-dimensionally preserved gut contents, general view and thin section; gut material (C) is easily distinguished from the matrix (D) by its brown colour due to high organic content. Crystals (in white) are not specific to the gut and are observed elsewhere in the matrix though smaller and less concentrated; they are interpreted as sponge spicules [21]. E, F, ROM 61755a, isolated fragment of gut content seen in transverse section. G, H, ROM 61754, gut contents showing small skeletal fragments and undetermined elements embedded in sediment. I, J, ROM 61755a, transverse section through upper part of gut content; the uppermost thin layer possibly represent the gut wall. K, ROM 61755b, thin carbonaceous film overlying gut contents, possibly representing the gut wall. gc, gut contents; se, sediment; sf, skeletal fragment; ue, undetermined element. A–D, courtesy L. Wilson (see also [21]). A, G are light photographs; B and D were taken in transmitted light; E, F, H-K are scanning electron micrographs (K, back-scattered image). Scale bar: 5 mm for A; 2 mm for G; 500 µm for B, H; 100 µm for C, D; 50 µm for K; 10 µm for E, I; 5 µm for F; 2 µm for J.

As with the majority of non-biomineralizing fossils from the Burgess Shale, *Ottoia prolifica* is preserved as compressed aluminosilicate and carbonaceous films [46], [47] (Fig. 2). *Ottoia* resembles Recent priapulids [48], [49] (Fig. 3) in having a retractile introvert armed with hooks and an invaginable pharynx lined with small teeth, two features of key-importance in locomotion and feeding [50]. The gut of *Ottoia* appears as a colored or reflective strip of constant width (1.4 to 2.3 mm in specimens 60–100 mm long [21]) running axially from the pharynx to the anus. It is either straight, sinuous or looped. EDX elemental mapping reveals anatomical partitioning of the gut with elevated C, Fe and P that probably reflects its organic-rich original composition and early diagenetic mineralizations in pyrite, apatite or calcite (Fig. 4). More than 50% of the studied specimens possess empty guts (Fig. 1) and about 20% display three-dimensionally preserved gut contents (GC) that preferentially concentrate in the posterior half of their digestive tract. GC typically occur as compacted ribbon-like features or fragmented blobs containing skeletal elements (e.g. hyolithid conchs, brachiopod valves), smaller debris of uncertain origin, and sediment. Thin section, SEM and EDX analyses do not show any significant compositional difference between GC and the aluminosilicate host rock, except from being enriched in organic matter (Fig. 5). Furthermore, acritarchs and sponge spicules found in comparable quantities in GC and the host rock [21] confirm that *Ottoia* ingested sediment.

#### (a) Hyolithids

**Figure 6:**
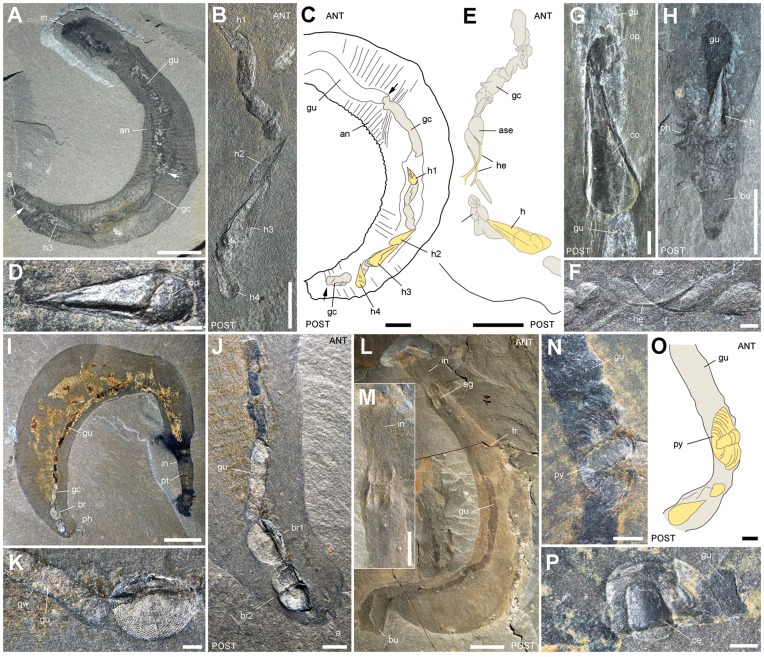
Hyolithids, brachiopods and arthropods within the gut of *Ottoia prolifica* from the middle Cambrian Burgess Shale. A–C, ROM 61747, with 4 hyolithid shells (*Haplophrentis carinatus*), their apex pointing anteriorly. D, ROM 61767, hyolithid with operculum and conch in connexion. E, F, ROM 61749, hyolithid conch and a pair of disarticulated helens. G, ROM 61774, hyolithid conch and disarticulated operculum. H, USNM 202777, hyolithid conch within the posteriormost part of the gut (bursa everted). I–K, ROM 61779 with two brachiopods (*Micromitra burgessensis*) in posterior gut. L, M, ROM 61775 with complete agnostid arthropod (*Ptychagnostus praecurrens*) within the anterior gut. N, O, ROM 61777 with a trilobite pygidium (*Ehmaniella burgessensis*) inside the gut. P, ROM 61785 with a trilobite cephalon (*E. burgessensis*). Abbreviations: a, anus; ag, agnostid; an, trunk annulation, ANT, anterior; ase, almond-shape element; br, brachiopod; br1, br2, from anterior, brachiopod 1 and 2; bu, bursa; ce, cephalon; co, hyolithid conch; gc, gut content; gu, gut; gw, gut wall; h1–h4, from anterior, hyolithid 1 to 4; he, helen; in, introvert; m, mouth; op, hyolithid operculum; ph, posterior hook; POST, posterior; pt, pharyngeal teeth; py, pygidium; tr, trunk. Scale bar: 1 cm for A, I, L; 5 mm for B, C, E, H, M; 2 mm for K; 1 mm for D, F, G, J, N–P.

**Figure 7:**
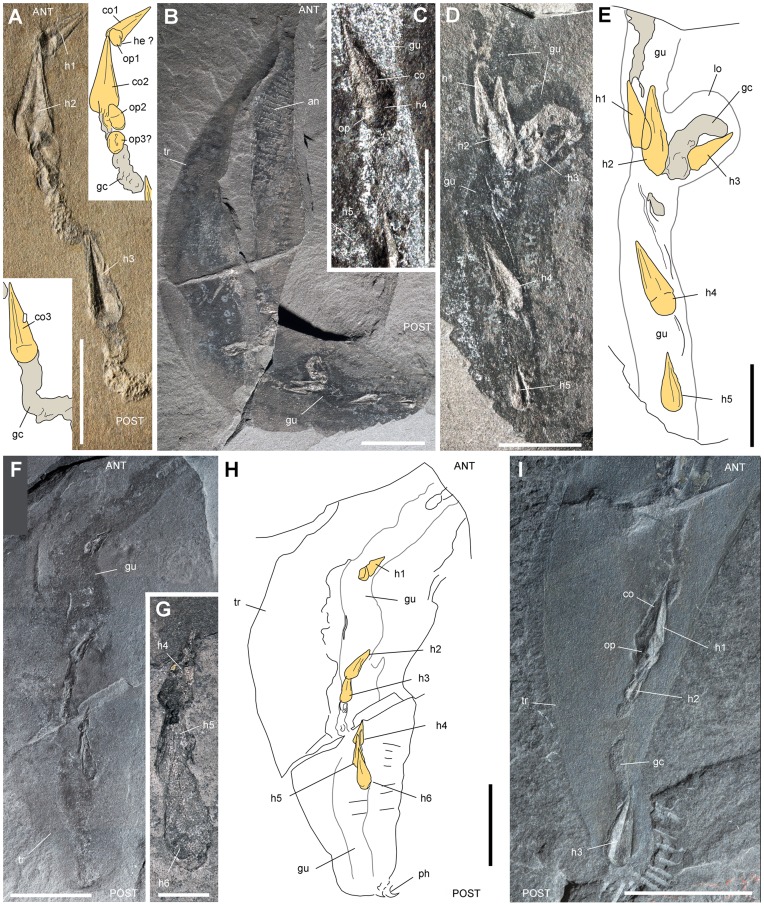
Hyolithids in the gut of *Ottoia prolifica* **from the middle Cambrian Burgess Shale.** A, ROM 61753a, showing 3 hyolithids preserved with their opercule. B–E, ROM 61782, with 5 hyolithid shells (*Haplophrentis carinatus*) within the gut, their apex pointing anteriorly; general view and details. F–H, USNM 196381, with 6 hyolithid shells within the gut. I, USNM 188604, with 3 hyolithids (h3 close to the anus). Abbreviations: ANT, anterior; an, trunk annulation; co1–co3, hyolithid conch 1 to 3; gc, gut content; gu, gut; he, helen; h1–h6, from anterior, hyolithid 1 to 6; op1–op3, hyolithid operculum 1 to 3; lo, loop; ph, posterior hook; POST, posterior; tr, trunk. Scale bar: 1 cm for B, F, H, I; 5 mm for A, C–E; 2 mm for G.

The most frequent animal in *Ottoia*’s GC (Fig. 1, Tables 1, 2, 3, 4 and Table S1) is the hyolithid *Haplophrentis carinatus* [1], [51] characterized by a mineralized exoskeleton with a pointed conch, an operculum and a pair of curved appendages called helens; Figs. 6A–H, 7). It occurs in 48% of GC that have identifiable elements (Fig. 1). The number of conchs varies from 1 to exceptionally 6; 82% of hyolithid-bearing GC have only 1 or 2 conchs; 62% of the conchs are ca. 3–6 mm long and 0.6–3 mm wide (Table 1). Hyolithids in GC are 3D-preserved and show no visible trace of physical breakdown or chemical dissolution, the conch and the operculum being sometimes connected (Fig. 6D). The very rare presence of helens within GC, either attached or detached from the conch, suggests that the majority of hyolithids became partly disarticulated as they entered the digestive tract of the worm (e.g. by the muscular contractions of pharynx). Helens may have been weakly attached in life, which may account for the low percentage (ca. 7%; [52]) of fully articulated hyolithids in the fossil assemblages. Hyolithid conchs show a remarkably consistent orientation with 77% of them pointing towards the mouth of *Ottoia*. This indicates that hyolithids were preferentially grasped and drawn into the gut by their anterior side, where they probably offered a stronger grip point to the pharyngeal teeth of *Ottoia.*

**Table 1:** Hyolithid elements in the gut contents of *Ottoia prolifica* from the Middle Cambrian Burgess Shale: countings and measurements.

HYOLITHIDS	RQ+RT	%	WQ+WT	%	USNM	%	ALL	%
***Ottoia prolifica*** ** with hyolithid conchs**	**53**	***100***	**19**	***100***	**28**	***100***	**100**	***100***
1 conch in gut	33	*62.5*	10	*53*	14	*50*	57	*57*
2 conchs in gut	10	*19*	7	*37*	8	*28.5*	25	*25*
3 conchs in gut	6	*11*	1	*5.5*	3	*11*	10	*10*
4 conchs in gut	3	*5.5*	0	*0*	2	*7*	5	*5*
5 conchs in gut	1	*2*	1	*5.5*	0	*0*	2	*2*
6 conchs in gut	0	*0*	0	*0*	1	*3.5*	1	*1*
**number of hyolithid conchs**	**88**	***100***	**32**	***100***	**53**	***100***	**173**	***100***
position 1 (anterior)	1	*1*	2	*6.5*	4	*7.5*	7	*4*
position 2 (mid-anterior)	8	*9*	3	*9.5*	6	*11.5*	17	*10*
position 3 (mid-posterior)	28	*58*	8	*25*	23	*43.5*	59	*34*
position 4 (posterior)	51	*32*	19	*59.5*	20	*37.5*	90	*52*
position 1 (anterior)	1	*1*	2	*6.5*	4	*7.5*	7	*4*
position 2 (mid-anterior)	8	*9*	3	*9.5*	6	*11.5*	17	*10*
position 3 (mid-posterior)	28	*58*	8	*25*	23	*43.5*	59	*34*
**orientation of conchs 1**	**72**	***82***	**24**	***75***	**37**	***70***	**133**	***77***
**orientation of conchs 2**	**16**	***18***	**8**	***25***	**16**	***30***	**40**	***23***
conch length: 0–0.99 mm	1	*1*	0	*0*	0	*0*	1	*0.5*
conch length: 1–1.99 mm	6	*7*	5	*16*	0	*0*	11	*6.5*
conch length: 2–2.99 mm	8	*9*	4	*13*	4	*8*	16	*9.5*
conch length: 3–3.99 mm	17	*19.5*	6	*19.5*	12	*23*	35	*20.5*
conch length: 4–4.99 mm	15	*17*	8	*26*	10	*19*	33	*19.5*
conch length: 5–5.99 mm	20	*23*	6	*19.5*	12	*23*	38	*22.5*
conch length: 6–6.99 mm	8	*9*	1	*3*	11	*21*	20	*11.5*
conch length: 7–7.99 mm	4	*4.5*	1	*3*	1	*2*	6	*3.5*
conch length: 8–8.99 mm	5	*6*	0	*0*	0	*0*	5	*3*
conch length: 9–9.99 mm	2	*2*	0	*0*	0	*0*	2	*1*
conch length: 10–10.99 mm	0	*0*	0	*0*	2	*4*	2	*1*

RQ, RT, WQ, WT: collections of the Royal Ontario Museum, Toronto, Raymond Quarry and talus, Walcott Quarry and talus, respectively. USNM, collections of the Smithsonian National Museum of Natural History, Washington D.C. Raw data in Table S1. orientation of conchs 1 = conch apex pointing upwards within the gut of *Ottoia*; orientation of conchs 2 = conch apex pointing downwards.

**Table 2:** Brachiopod elements in the gut contents of *Ottoia prolifica* from the Middle Cambrian Burgess Shale: countings and measurements.

BRACHIOPODS	RQ+RT	%	WQ+WT	%	USNM	%	ALL	%
***Ottoia prolifica*** ** with brachiopods**	**15**	***100***	**2**	***100***	**1**	***100***	**18**	***100***
with *Micromitra burgessensis*	10	*67*	1	*50*	0	*0*	11	*61*
with *Diraphora bellicostata*	1	*7*	0	*0*	1	*100*	2	*11*
with undet. brachiopods	4	*26*	1	*50*	0	*0*	5	*18*
**number of brachiopod valves**	**18**	***100***	**2**	***100***	**1**	***100***	**21**	*100*
position 1 (anterior)	**0**	***0***	**0**	***0***	**0**	***0***	**0**	*0*
position 2 (mid-anterior)	1	*5.5*	0	*0*	1	*100*	2	*9.5*
position 3 (mid-posterior)	6	*33.5*	0	*0*	0	*0*	6	*28.5*
position 4 (posterior)	11	*61*	2	*100*	0	*0*	13	*62*
**number of measured valves**	**18**	***100***	**2**	***100***	**1**	***100***	**21**	***100***
valve width: 0–0.99 mm	1	*5.5*	1	*50*	0	*0*	2	*9,5*
valve width: 1–1.99 mm	6	*33.5*	1	*50*	0	*0*	7	*33.5*
valve width: 2–2.99 mm	6	*33.5*	0	*0*	0	*0*	6	*28.5*
valve width: 3–3.99 mm	3	*16.5*	0	*0*	0	*0*	3	*14*
valve width: 4–4.99 mm	0	*0*	0	*0*	1	*100*	1	*5*
valve width: 5–5.99 mm	2	*11*	0	*0*	0	*0*	2	*9.5*
valve width: 6–6.99 mm	0	*0*	0	*0*	0	*0*	0	*0*

RQ, RT, WQ, WT: collections of the Royal Ontario Museum, Toronto, Raymond Quarry and talus, Walcott Quarry and talus, respectively. USNM, collections of the Smithsonian National Museum of Natural History, Washington D.C. Raw data in Table S1.

**Table 3:** Arthropod elements in the gut contents of *Ottoia prolifica* from the Middle Cambrian Burgess Shale: countings and measurements.

3-BRADORIIDS	RQ+RT	%	WQ+WT	%	USNM	%	ALL	%
***Ottoia prolifica*** ** with agnostids**	**6**	***100***	**9**	***100***	**1**	***100***	**17**	***100***
with *Pagetia bootes*	5	*62.5*	2	*40*	1	*25*	9	*47*
with *Ptychagnostus praecurrens*	2	*25*	2	*40*	0	*0*	3	*23.5*
with undet. agnostids	1	*12.5*	1	*20*	3	*75*	5	*29.5*
**number of agnostid elements**	**8**	***100***	**6**	***100***	**6**	***100***	**20**	***100***
position 1 (anterior)	2	*25*	1	*16.5*	0	*0*	3	*15*
position 2 (mid-anterior)	1	*12.5*	0	*0*	1	*16.5*	3	*15*
position 3 (mid-posterior)	1	*12.5*	3	*50*	1	*16.5*	10	*50*
position 4 (posterior)	4	*50*	2	*33.5*	4	*67*	4	*20*
**number of measured agnostid elements**	**8**	***100***	**5**	***100***	**2**	***100***	**15**	***100***
width: 0–0.99 mm	1	*12.5*	0	*0*	0	*0*	1	*6.5*
width: 1–1.99 mm	4	*50*	1	*20*	0	*0*	5	*33.5*
width: 2–2.99 mm	3	*37.5*	1	*20*	0	*0*	4	*26.5*
width: 3–3.99 mm	0	*0*	1	*20*	2	*100*	3	*20*
width: 4–4.99 mm	0	*0*	2	*40*	0	*0*	2	*13.5*
width: 5–5.99 mm	0	*0*	0	*0*	0	*0*	0	*0*
width: 6–6.99 mm	0	*0*	0	*0*	0	*0*	0	*0*
**2- TRILOBITES**	**RQ+RT**	**%**	**WQ+WT**	**%**	**USNM**	**%**	**ALL**	**%**
***Ottoia prolifica*** ** with trilobites**	**1**	***100***	**5**	***100***	**2**	***100***	**8**	***100***
with *Ehmaniella waptensis*	0	*0*	2	*40*	0	*0*	2	*25*
with undet. trilobites	1	*100*	3	*60*	2	*100*	6	*75*
**number of trilobite elements**	**1**	***100***	**6**	***100***	**2**	***100***	**9**	***100***
position 1 (anterior)	0	*0*	1	*16.5*	0	*0*	0	*0*
position 2 (mid-anterior)	0	*0*	0	*0*	1	*50*	3	*33.5*
position 3 (mid-posterior)	1	*100*	1	*16.5*	0	*0*	2	*22*
position 4 (posterior)	0	*0*	4	*67*	1	*50*	4	*44.5*
**number of measured trilobite elements**	**1**	***100***	**5**	***100***	**0**	***0***	**6**	***100***
width: 0–0.99 mm	0	*0*	0	*0*	0	*0*	0	*0*
width: 1–1.99 mm	1	*100*	1	*20*	0	*0*	2	*33.3*
width: 2–2.99 mm	0	*0*	0	*0*	0	*0*	0	*0*
width: 3–3.99 mm	0	*0*	2	*40*	0	*0*	2	*33.3*
width: 4–4.99 mm	0	*0*	2	*40*	0	*0*	2	*33.3*
width: 5–5.99 mm	0	*0*	0	*0*	0	*0*	0	*0*
width: 6–6.99 mm	0	*0*	0	*0*	0	*0*	0	*0*
**3- BRADORIIDS**	**RQ+RT**	***%***	**WQ+WT**	***%***	**USNM**	***%***	**ALL**	***%***
***Ottoia prolifica*** ** with bradoriids**	**6**	***100***	**9**	***100***	**1**	***100***	**17**	***100***
**number of bradoriid elements**	**6**	***100***	**11**	***100***	**1**	***100***	**22**	***100***
position 1 (anterior)	1	*17*	2	*18*	0	*0*	4	*18*
position 2 (mid-anterior)	2	*33*	2	*18*	1	*100*	4	*18*
position 3 (mid-posterior)	1	*17*	4	*36.5*	0	*0*	6	*27.5*
position 4 (posterior)	2	*33*	3	*27.5*	0	*0*	8	*36.5*
**number of measured valves/carapaces**	**6**	***100***	**11**	***100***	**1**	***100***	**20**	***100***
valve/carapace length: 0–0.99 mm	0	*0*	1	*9*	0	*0*	1	*5*
valve/carapace length: 1–1.99 mm	5	*83.5*	7	*64*	1	*100*	16	*80*
valve/carapace length: 2–2.99 mm	1	*16.5*	3	*27*	0	*0*	3	*15*
valve/carapace length: 3–3.99 mm	0	*0*	0	*0*	0	*0*	0	*0*
valve/carapace length: 4–4.99 mm	0	*0*	0	*0*	0	*0*	0	*0*
valve/carapace length: 5–5.99 mm	0	*0*	0	*0*	0	*0*	0	*0*
valve/carapace length: 6–6.99 mm	0	*0*	0	*0*	0	*0*	0	*0*

RQ, RT, WQ, WT: collections of the Royal Ontario Museum, Toronto, Raymond Quarry and talus, Walcott Quarry and talus, respectively. USNM, collections of the Smithsonian National Museum of Natural History, Washington D.C. Raw data in Table S1.

**Table 4:** Almond-shape elements (ASE; see Fig. 12) in the gut contents of *Ottoia prolifica* from the Middle Cambrian Burgess Shale: countings and measurements.

ASE	RQ+RT	*%*	WQ+WT	*%*	USNM	*%*	ALL	*%*
***Ottoia prolifica*** ** with ASE**	**5**	***100***	**13**	***100***	**4**	***100***	**22**	***100***
**number of ASE**	**15**	***100***	**66**	***100***	**26**	***100***	**107**	***100***
position 1 (anterior)	0	*0*	0	*0*	0	*0*	0	*0*
position 2 (mid-anterior)	0	*0*	0	*0*	1	*4*	1	*1*
position 3 (mid-posterior)	6	*40*	16	*24*	9	*34.5*	31	*29*
position 4 (posterior)	9	*60*	50	*76*	16	*61.5*	75	*70*
**number of measured ASE**	**13**	***100***	**45**	***100***	**16**	***100***	**74**	***100***
length: 1–1.49 mm	0	*0*	0	*0*	0	*0*	0	*0*
length: 1.5–1.99 mm	0	*0*	6	*13*	0	*0*	6	*8*
length: 2–2.49 mm	2	*15*	7	*15.5*	2	*12.5*	11	*15*
length: 2.5–2.99 mm	3	*23*	9	*20*	4	*25*	16	*22*
length: 3–3.49 mm	5	*38*	10	*22*	5	*31*	20	*27*
length: 3.5–3.99 mm	1	*8*	4	*9*	2	*12.5*	7	*9.5*
length: 4–4.99 mm	1	*8*	2	*4.5*	2	*12.5*	5	*7*
length: 5–5.49 mm	0	*0*	3	*7*	0	*0*	3	*4*
length: 5.–5.49 mm	0	*0*	2	*4.5*	0	*0*	2	*2.5*
length: 5.5–5.99 mm	1	*8*	2	*4.5*	1	*6.5*	4	*5*
length: 6. 6.49 mm	0	*0*	0	*0*	0	*0*	0	*0*

RQ, RT, WQ, WT: collections of the Royal Ontario Museum, Toronto, Raymond Quarry and talus, Walcott Quarry and talus, respectively. USNM, collections of the Smithsonian National Museum of Natural History, Washington D.C. Raw data in Table S1.

#### (b) Brachiopods

**Figure 8:**
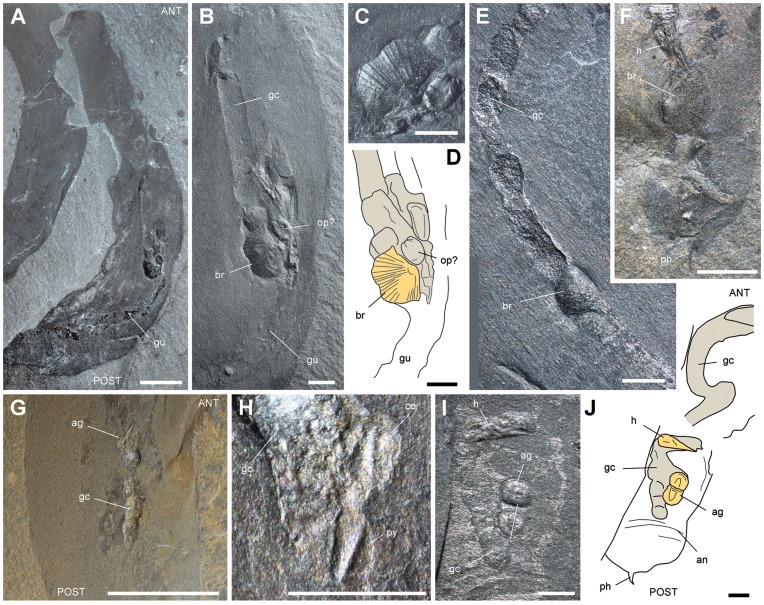
Other skeletal elements in the gut of *Ottoia prolifica* **from the Middle Cambrian Burgess Shale.** A–D, USNM 196204, with articulate brachiopod, possibly *Diraphora bellicostata* [1], [50], [51]. E, ROM 61756, with the inarticulate brachiopod *Micromitra burgessensis* [1], [50], [51] and undetermined gut contents. F, ROM 61750 with *Micromitra burgessensis* and a hyolithid. G, H, ROM 61770 with agnostid (possibly *Pagetia bootes* [1], [52]), general view and close-up. I, J, ROM 61783 with complete agnostid and hyolithid. Abbreviations: ag, agnostid; an, trunk annulation; ANT, anterior; br, brachiopod; ce, cephalon; gc, gut contents; gu, gut; h, hyolithid; op, operculum of hyolithid; ph, posterior hook; POST, posterior; py, pygidium. All light photographs. Scale bar: 1 cm for A, G; 2 mm for B–F, H–J.

Articulate brachiopods (Table 2) are represented in GC by *Micromitra burgessensis* [1], [53], [54] characterized by a very distinctive lozenge-like reticulated pattern (Figs. 6I–K, 8A–F) and, possibly *Diraphora* [1], [53], [54], although much more rarely. The best-preserved specimens of *Micromitra burgessensis* (not in GC) are fringed with long and delicate setae which indicates that the animal did not live buried in the sediment [1] but more likely at the water sediment interface.

#### (c) Arthropods

**Figure 9:**
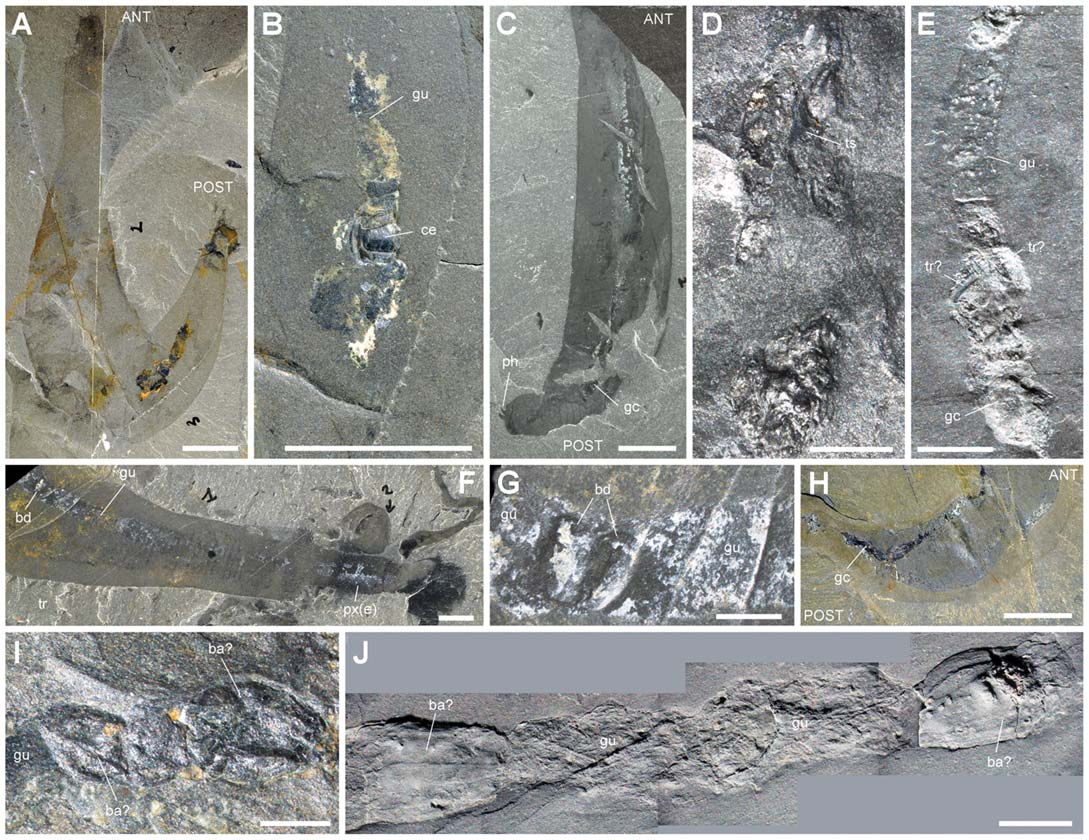
Other skeletal elements in the gut of *Ottoia prolifica* **from the middle Cambrian Burgess Shale.** A, B, ROM 61785, showing gut contents with a trilobite cephalon (probably *Ehmaniella burgessensis* [1], [52]). C, D, ROM 61761, with contents containing trilobite remains (e.g. thoracic segments). E, USNM 196425, with gut contents containing possible trilobite remains. F, G, ROM 61776, with bradoriid arthropod [53] in anterior part of gut. H, I, ROM 61778, with possible shields of bivalved arthropods in posterior gut, general view and detail. J, ROM 61771, with possible shields of bivalved arthropods. Abbreviations: ANT, anterior; ba, bivalved arthropod (shield); bd, bradoriid; ce, cephalon; gc, gut contents; gu, gut; ph, posterior hook; POST, posterior; px(e), everted pharynx; tr, trilobite; ts, thoracic segment. All light photographs (J, whitened with ammonium chloride). Scale bar: 1 cm for A–C; 5 mm for F; 2 mm for D, E, G; 1 mm for I, J.

Arthropod skeletal elements (Table 3) are frequent, represented mainly by agnostids, small trilobites and bradoriids (Figs. 6L–P; 8G–J). The agnostids *Ptychagnostus praecurrens* [1], [55] and possibly *Pagetia bootes* [1], [55] occur as mainly disarticulated exoskeletal elements (anterior and posterior shields, thoracic segments), except for one complete specimen found within the anterior-most section of the gut just behind the pharynx (Fig. 6L, M). The trilobite *Ehmaniella* [1], [55] is represented by isolated cephalons, pygidia and disarticulated thoracic segments (Figs. 6N–P, 9A–E). The bradoriid *Liangshanella burgessensis* [56] is a tiny arthropod capped by a dorsally folded shield. Although extremely abundant in the Burgess Shale biota [2], *L. burgessensis* is a rare element in GC (Fig. 9F, G). Indeterminate bivalved arthropods different from bradoriids also occur as shield-like folded features (Fig. 9I, J). In addition to these readily identifiable undigested remains are setae-like (SLE) and almond-shape (ASE) skeletal elements.

#### (d) Setae-like elements (SLE)

**Figure 10:**
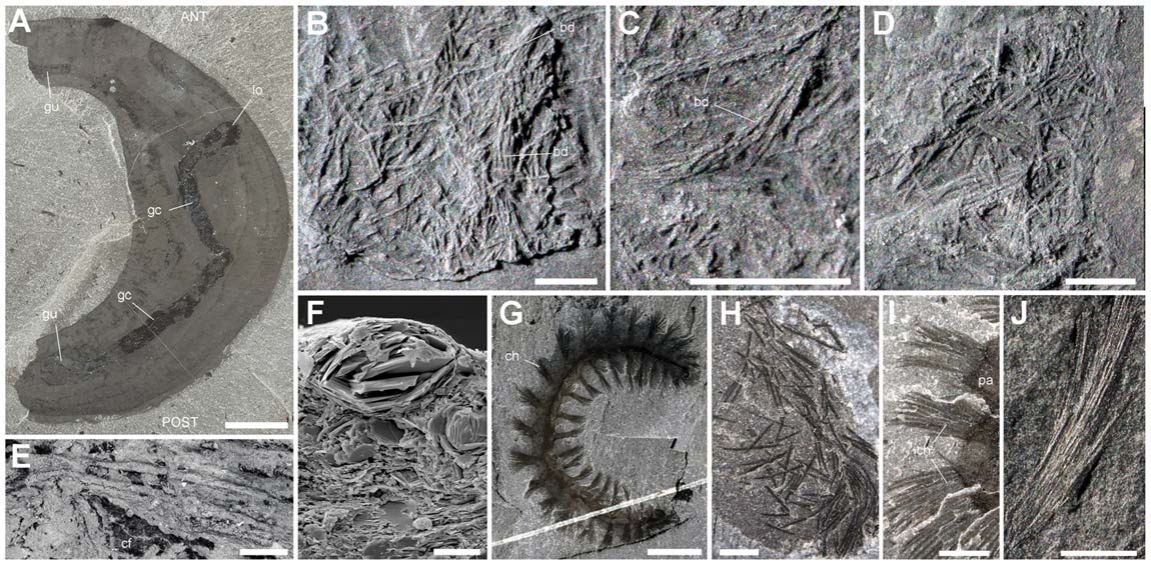
Setae-like elements (SLE) within the gut of *Ottoia prolifica* from the middle Cambrian Burgess Shale, compared with the chaetae of *Burgessochaeta*. A–D, ROM 61755b, general view, accumulations and details of SLE in gut. E, ROM 61772b, SLE in gut. F, ROM 61746a, SLE in cross section, preserved in aluminosilicate. G–I, *Burgessochaeta setigera* (Polychaeta; [1], [54]); G, ROM 56967 complete specimen with numerous chaetae on parapodia; H, ROM 56968a(1), ROM 56968a(1), decayed specimen; I, ROM 56968a(2), chaetae on parapodia. J, ROM 56969a, bundle of chaetae (compare with C). Abbreviations: ANT, anterior; bd, possible bundle of SLE; cf, carbonaceous film; ch, chaetae; gc, gut content; gu, gut; pa, parapodium; POST, posterior. Scale bar: 1 cm for A, G; 1 mm for E, H–J; 500 µm for B; 100 µm for D; 20 µm for F.

**Figure 11:**
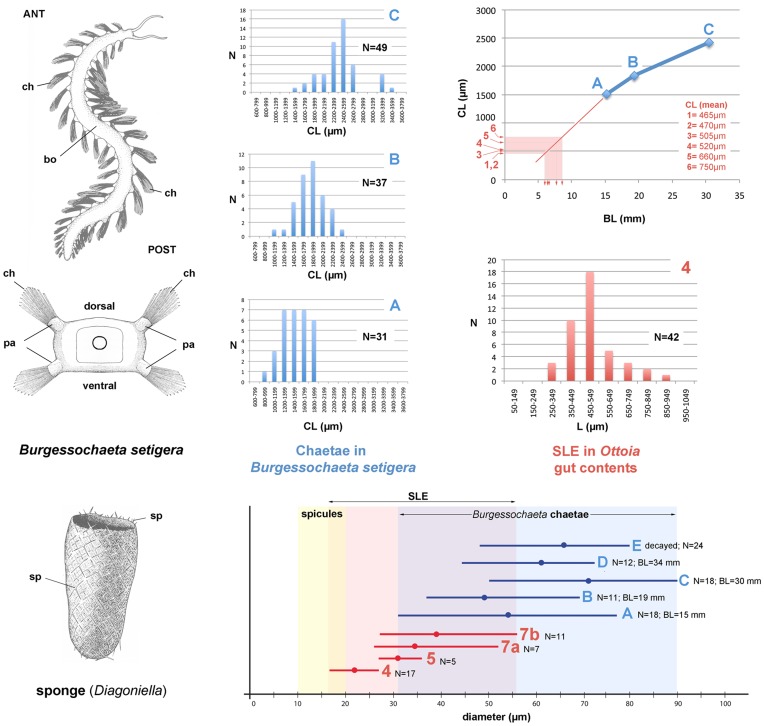
Comparative measurements between the setae-like elements (SLE) within the gut of *Ottoia prolifica,* the chaetae of *Burgessochaeta setigera* [**1**], [**59**] **and sponge spicules.** The diameter of most spicules of sponges occurring in the same horizons as *Ottoia* ranges between 10 and 20 µm and is lower than that of most SLE. Interpolation (up right diagram) suggests that SLE are undigested chaetae of small individuals of *Burgessochaeta,* possibly between 5 and 10 mm long. Size distribution of chaetae (blue) and SLE (red) lengths are given for three well-preserved *Burgessochaeta* specimens (A–C) and one *Ottoia* gut content (number 4). *Diagonella* (bottom left) is a typical sponge in the Burgess Shale biota. Abbreviations: ANT, anterior; BL, body length; bo, body; ch, chaetae; CL, chaeta length; pa, parapodium; POST, posterior; sp, spicule. 1, ROM 61786b; 2, ROM 61787; 3, ROM 61772b; 4, ROM 61755; 5, ROM 61788; 6, ROM 61789; 7, ROM 61746. A, ROM 56968a; B, ROM 56968b; C, ROM 56967; D, ROM 56968b; E, ROM 56969a.

SLE generally occur as large concentrations of straight or slightly curved 3D-preserved cylindrical elements (Fig. 10). Their size (length and diameter 50–950 and 17–55 µm, respectively; Fig. 11) is not consistent with a sponge origin (Figs. 11, Fig. S1). Most sponges occurring in the same horizon or associated with *Ottoia* on the same bedding plane [57], [58] have monaxial needle-like elements (diameter between 10 and 20 µm) usually tightly clustered to form tracts or tufts. *Pirania* has strong radial spicules (length >7 mm and diameter >100 µm). No cross-shaped or rayed structure typical of hexactinellid (e.g. *Protospongia*) or stem-group calcareous (e.g. *Eiffelia*) sponges was found in SLE. That SLE are arthropod setae is unlikely because of the lack of tergites, shields or appendages associated with them. SLE are interpreted as the chaetae of the polychaete worm *Burgessochaeta setigera* [1], [59] (Figs. 10G–J, 11) that effectively co-occurs with *Ottoia* (Table 5). Supporting evidence comes from the high number of chaetae in *Burgessochaeta* (>1000 attached to more than 20 pairs of biramous parapodia), their size range (diameter 30–90 µm) and frequent groupings in bundles (Fig. 10B–E). The size of SLE is consistent with *Ottoia* feeding on juveniles of *Burgessochaeta* (Fig. 11). Polychaete chaetae are frequent in the feces of Recent priapulid worms such as *Priapulus* (Fig. 3I).

**Table 5:** Numerical abundance of *Ottoia prolifica* and the animal taxa that constituted its diet (evidence from gut contents and feeding assemblages, present paper) through successive bed assemblages in the Great Phyllopod Bed (Walcott Quarry Member, Burgess Shale Formation, Middle Cambrian).

BA	nb. ind.	nb. taxa	A	B(1)	C(2)	D(3)	E(4)	F(5)	G(5)	H(5)	I(6)	J(7)	K
**+120**	516	43	**11**	8	0	1	23	21	10	4	1	0	2
**0**	1423	55	**6**	2	14	1	53	11	24	0	0	0	3
**−40**	229	26	**1**	1	9	3	10	7	0	0	0	0	0
**−110**	585	53	**6**	4	3	1	134	3	38	194	0	1	10
**−**120	3312	92	**272**	8	54	1	165	4	106	164	16	16	12
**−130**	3267	79	**27**	4	5	1	107	2	156	7	5	6	44
**−150**	2930	85	**3**	27	0	1	326	35	757	106	9	44	54
**−170**	1488	73	**10**	8	0	28	47	10	393	4	11	5	9
**−210**	4609	105	**35**	29	0	31	274	18	1011	65	54	12	139
**−220**	93	28	**1**	0	0	5	6	1	2	0	1	0	1
**−235**	2247	84	**63**	12	0	51	284	4	175	33	6	5	38
**−245**	4614	62	**3**	13	0	44	1400	14	238	248	1	13	54
**−250**	2478	74	**12**	8	0	7	833	7	91	98	2	8	18
**−260**	3844	79	**46**	10	25	25	1079	19	203	51	2	19	76
**−265**	1842	70	**22**	2	2	2	414	9	140	11	4	3	25
**−270**	216	33	**3**	0	0	1	28	2	4	2	0	0	0
**−310**	915	63	**4**	3	0	15	49	16	115	1	1	1	21
**−315**	189	22	**0**	0	0	1	3	23	30	0	0	2	11
**−320**	1561	66	**16**	5	1	14	11	12	64	1	2	1	10
**−350**	4258	40	**44**	0	2	100	13	14	22	0	1	0	1
**−355**	233	27	**0**	1	2	38	6	6	57	1	0	1	6
**−360**	2392	43	**2**	1	2	4	29	21	74	1	4	2	1
**−370**	582	48	**0**	3	0	3	19	26	76	21	0	3	14
**−380**	455	38	**9**	0	9	5	69	4	20	2	5	0	1
**−400**	2548	92	**56**	7	32	29	65	23	41	2	12	1	62
**−410**	172	32	**1**	1	0	1	3	1	6	0	0	0	3
**−418**	115	18	**1**	0	0	1	29	1	1	1	0	0	0
**−420**	1570	40	**12**	8	9	2	475	3	10	0	1	1	14
**−430**	430	31	**1**	1	0	7	40	2	15	2	1	0	6
**−445**	1563	41	**0**	1	0	5	98	41	72	1	1	2	13
**−455**	404	29	**1**	0	11	31	13	5	0	0	1	1	2
**−465**	686	23	**0**	7	0	1	13	5	19	2	0	6	9
**−480**	381	59	**8**	4	2	1	25	13	8	0	1	6	1
**−495**	101	24	**1**	1	6	1	9	5	0	0	0	0	0
**−500**	192	27	**0**	7	0	0	33	3	7	0	1	1	4
**−502**	180	25	**0**	0	1	2	15	1	0	0	1	0	0

Faunal data courtesy J.-B. Caron and [2], [42], [52].

*A, Ottoia prolifica* (Priapulida); *B, Haplophrentis carinatus* (hyolithid); *C, Burgessochaeta setigera* (Polychaeta); *D, Wiwaxia corrugata* (wiwaxiid); *E, Liangshanella burgessensis* (bradoriid arthropod); *F, Ehmaniella ssp.* (Trilobita); *G, Ptychagnostus praecurrens* (agnostid arthropod); *H, Pagetia bootes* (agnostid arthropod); *I, Sidneyia inexpectans* (Arthropoda); *J, Mitromitra burgessensis* (Brachiopoda); *K, Diraphora bellicostata* (Brachiopoda).

(1) including individual shell operculum or shell whichever is greater; (2) all collected specimens; (3) excluding isolated sclerites. Count of one specimen when presence of isolated remains only (levels: 120, **−**110, **−**130, **−**150, **−**270, **−**315, **−**418, **−**465, **−**495); (4) number of specimens without soft tissues divided by two to compensate for the presence of dissociated valves;(5) including number of cephala or pygidia whichever is greater; (6) excluding isolated thoracic tergites. Count of one specimen when presence of isolated remains only (levels: 120, **−**350, **−**430, **−**445, **−**455, **−**500, **−**500); (7) excluding fragments of shells (exception **−**500 with a single occurrence).

#### (e) Almond-shape elements (ASE)

**Figure 12:**
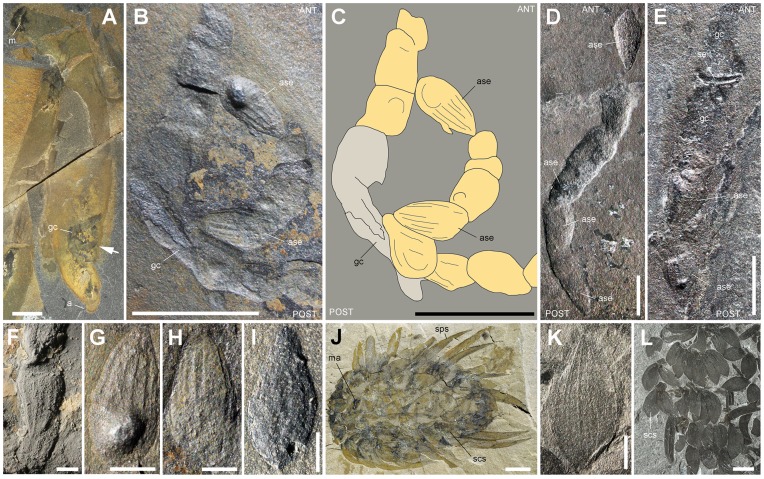
Almond-shape elements (ASE) within the gut of *Ottoia prolifica* from the middle Cambrian Burgess Shale, compared to the sclerites of *Wiwaxia* [**60**] **.** A–C, G, H, ROM 61768, general views, and details of ASE (bulbous feature in G is an artefact due to mineralization). D, ROM 61763b with aligned ASE. E, ROM 61773a, with ASE and other skeletal elements. F, ROM 61781a, three aligned ribbed ASE. I, ROM 61745b, isolated ASE within gut. J, ROM 61747, *Wiwaxia corrugata* [1], [60] with sclerites in situ. K, L, ROM 56965, *W. corrugata,* ribbed sclerite and general view of decayed specimen. Abbreviations: a, anus; ANT, anterior; ase, almond-shape element; gc, gut content; m, mouth; ma, mouth apparatus; POST, posterior; scs, scale-like sclerite; se, skeletal element; sps, spine-like sclerite. Scale bar: 5 mm for A–C, J, L; 2 mm for D, E, K; 1 mm for F–I.

ASE (Table 4) have a consistent almond shape, are slightly convex, and bear at least 6 ribs parallel to their margins (Fig. 12). They typically occur in GC as aligned elements (N = 1 to 12; 34% over 9; Table 1) often overlapping each other. Their length varies from 1.5 to 6 mm (63% between 2 and 3.5 mm). More than 88% of ASE point towards the anus of *Ottoia -* i.e. - the opposite direction of hyolithid shells in GC (compare with Figs. 6A–H, 7). The only skeletal elements comparable in size, shape and ornament with ASE are the scale-like sclerites of wiwaxiids, especially those of *Wiwaxia corrugata* [1], [60] (Fig. 12J) that co-occurs with *Ottoia* (Table 5). The relatively low number of ASE in GC, the absence of typical spiny and crescentic elements, and the average size of *Wiwaxia* (>20 mm vs. gut diameter of *Ottoia* <3 mm) is not consistent with wiwaxiids being ingested whole by *Ottoia*. More likely it suggests that *Ottoia* fed on decaying wiwaxiids by ingesting lumps of soft tissues where small sclerites were still attached. The consistent orientation of ASE in GC may be explained by both the unidirectional imbricated pattern of the *Wiwaxia* scleritome [60] and also by capture constraints (see hyolithids). The cannibalistic behavior of *Ottoia* based on a single poorly preserved specimen [35] is not confirmed here although this behavior clearly remains plausible (see recent priapulid worms such as *Priapulus*; [61]). I re-examined this specimen (USNM 198922). The spinules and proboscis hooks that are assumed to be present within its gut are most probably preservational artefacts or due to the chance juxtaposition of two ill-preserved *Ottoia* specimens as suggested by L. Wilison [21]. Gut contents from the Raymond Quarry are largely dominated by hyolithids, whereas SLE (assumed polychaetes), hyolithids and ASE (assumed wiwaxiids) prevail in GC from the Walcott Quarry (Fig. 1). This suggests that *Ottoia* was not dependent on one particular food source but could adapt its diet with local food availability.

### Fossil Associations

**Figure 13:**
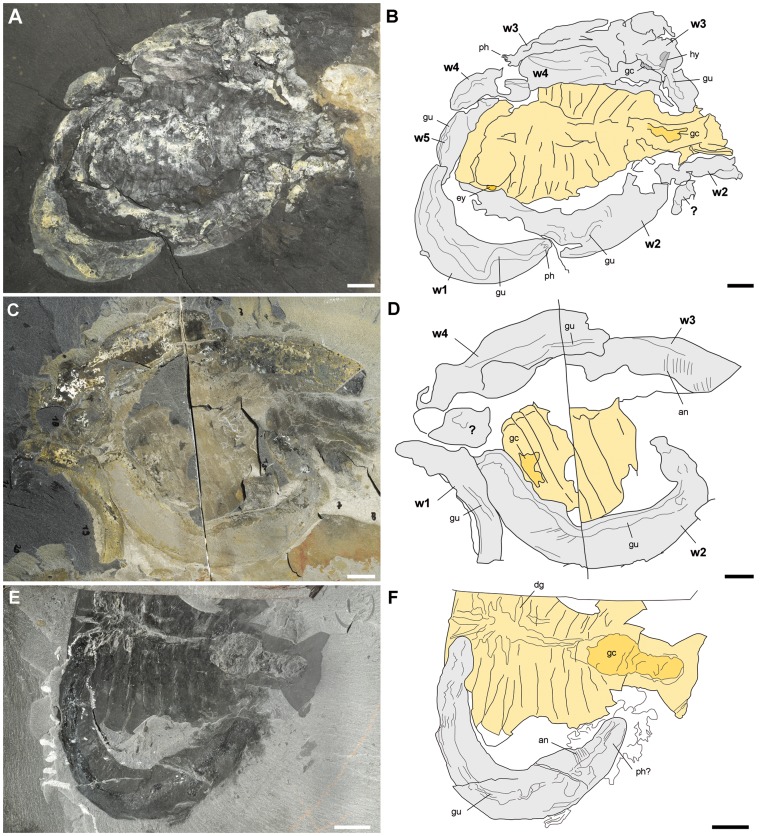
Three fossil associations from the Burgess Shale Formation, middle Cambrian, showing *Ottoia prolifica* around and below the carcass of the arthropod *Sidneyia inexpectans* and suggesting scavenging behaviour in *Ottoia prolifica.* A, B, USNM 196241, showing at least 5 worms around the decaying carcass of *Sidneyia*. This specimen was interpreted [32] as a death assemblage with the worms feeding around the collapsed and decaying carcass of *Sidneyia inexpectans*. I follow this interpretation here, although the number of worms is more likely to be five than nine [32]. C, D, ROM 61748a, showing an assemblage very similar to USNM 196241; four worms form a wreath-like arrangement around the remains of *Sidneyia*. E, F, USNM 250218, showing a curved specimen of *Ottoia* closely associated with *Sidneyia*. All light photographs (A, courtesy Jean-Bernard Caron, ROM). Scale bar: 1 cm. Abbreviations: an, annulation; dg, digestive glands; gc, gut content; gu, gut; hy, hyolithid conch; ph, posterior hook; px, pharynx; w1–5, worm (*Ottoia*) 1–5.

Two fossil associations with several specimens of *Ottoia* forming a wreath around the carcass of the arthropod *Sidneyia* [31], [32] indicate that *Ottoia* had possible scavenging habits (Fig. 13). Decaying carcasses of relatively large epibenthic animals such as *Sidneyia* (length up to ca 140 mm [31]) may have represented a substantial food source for *Ottoia*, easily accessible from its supposed shallow subhorizontal burrows [50]. The tiny pharyngeal teeth of *Ottoia* (Fig. 2E, F) are interpreted as a possible adaptation for scraping soft material such as decaying tissues.

## Discussion

### Feeding Process

**Figure 14:**
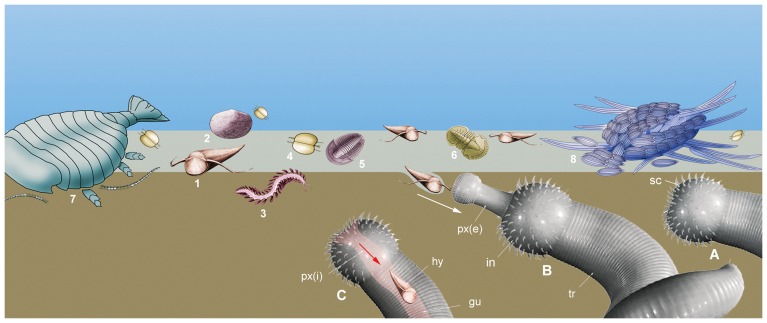
Major components of the diet of *Ottoia prolifica* **from the middle Cambrian Burgess Shale.** 1, hyolithids (*Haplophrentis*); 2, brachiopods (*Micromitra*); 3, polychaete worms (*Burgessochaeta*); 4, bradoriids (*Liangshanella*); 5, trilobites (*Ehmaniella*); 6, agnostids (*Ptychagnostus*); 7, 8, carcasses of *Sidneyia and Wiwaxia.* (A–C), feeding behavior of *Ottoia*: detection of food via possible chemical cues and ingestion. gu, gut; in, introvert with spiny scalids; px(e), everted pharynx; px(i), inverted pharynx; sc, scalid; tr, trunk.

The feeding mechanism of *Ottoia* was remarkably simple, being limited to the transit of food via a tubular gut with no physical breakdown (except the disarticulation of composite exoskeletons) and storage process. Nutrients were probably chemically extracted from food via digestive fluids produced in the midgut lumen as in Recent priapulids [48]. The assumed low nutritional value of some of the food items such as hyolithids, brachiopods that probably contained less protein-rich tissues than arthropods and worms; [62]) may have been offset by the richer intake of dead tissues from carcasses (Fig. 13). *Ottoia* lacked visual and complex sensory organs, in contrast with the arthropods from the same horizons that had potential features (e.g. compound eyes, antennae) for visual [10] and chemo-tactile recognition. Attraction to food was probably triggered by chemical cues released from living and dead tissues (Fig. 14A). Chemoreceptors were possibly located in the well-developed circumoral scalids (Fig. 2G), as is the case in modern priapulids worms ([63] and Fig. 3E, G).

### Trophic Complexity of the Cambrian Ecosystem

*Ottoia* obtained food from diverse animal sources (nine species in GC) and by using different techniques: 1) predation on small invertebrates that lived at or near the water-sediment interface (e.g., hyolithids, brachiopods, and polychaetes); and 2) scavenging on carcasses and detritus. The brachiopods and hyolithids from the Burgess Shale biota were most probably slow moving animals that could have been equally ingested alive or scavenged after death by *Ottoia*. No fossil evidence indicates that *Ottoia* favored predation over scavenging or the reverse. In contrast, polychaetes such as *Burgessochaeta* were probably far more active errant and burrowing animals with capabilities to escape predators such as *Ottoia*. Again, *Ottoia* may have fed indiscriminately upon dead and living polychaetes in various proportion depending on its hunting abilities and the rapidity of the prey. The idea that hyolithids were “hunted” [39] may not reflect the exact reality of feeding relationships. More likely these small invertebrates that often lived in large populations were taken off randomly by *Ottoia* which may have lived in sub-horizontal burrows just below the water sediment interface [50]. The presence of disarticulated elements in GC, typically trilobites, cannot be interpreted as unambiguous evidence of predation, because it may result from chance ingestion during scavenging. Similarly, fine sediment was inevitably ingested along with consumable food. The high percentage of empty guts indicates that *Ottoia* was neither a sediment eater *sensu stricto* nor a continuous feeder. Its straight cylindrical gut is also poorly consistent with continuous deposit feeding exemplified by modern sipunculans [64]. The gut of Recent and Cambrian [65] sipunculans is typically U-shaped and highly coiled. Although we cannot exclude that *Ottoia* collected and ingested undifferentiated particles and detritus (as possibly indicated by the organic enrichment of GC), this worm had none of the characteristics of a surface deposit feeder (e.g., introvert with small tentacles). Moreover, the ratio of its body to gut volume (0.8–1.5%) [21] is much lower than in typical deposit feeders. *Ottoia* was more likely an intermittent omnivorous feeder with low maintenance requirements. Possible modern analogues are macrobenthic priapulids such as *Priapulus* and *Halicryptus* [66], [67], in which guts are frequently empty and contain detritus mixed with identifiable animal food items (Table 6; [66]–[68]). Our study undermines the status of *Ottoia* as an iconic predator and selective hunter [39] and gives this taxon the more realistic status of being a generalist and possibly facultative feeder [69] – i.e., an animal with the capacity to vary its diet with local availability. In recent marine ecosystems, facultative feeders play an important role in conferring resilience in the benthic communities to environmental disturbances and habitat changes [69]. *Ottoia* may have played a comparable and important role at a critical time when the first modern-style ecosystems started to build up.

**Table 6:** Diet of Recent macrobenthic priapulid worms exemplified by *Priapulus caudatus* and *Halicryptus spinulosus* (see morphology in Fig. 3).

Diet of *Priapulus caudatus*	higher taxa 1	higher taxa 2	source of data	refs
*Aphrodite*	Annelida	Polychaeta	feces	[61]
*Amphiura chiaji*	Echinodermata	Ophiurida	feces; feeding exp.	[61]
*Terrebellides strömi*	Annelida	Polychaeta	feeding exp.	[61]
*Mellina costata*	Annelida	Polychaeta	feeding exp.	[61]
*Amphicteis gunneri*	Annelida	Polychaeta	feeding exp.	[61]
*Priapulus caudatus* (cannibalism)	Priapulida	–	live observations	[61]
*Priapulus caudatus* (cannibalism)	Priapulida	–	live observations	[68]
*Saccoglossus kowalewskyi* (fragment)	Hemichordata	Enteropneusta	feeding exp.	[68]
*Cerebratulus marginatus* (fragment)	Nemertea	–	feeding exp.	[68]
algal remains	–	–	gut contents	[61]
mud and unrecognizable debris	–	–	feces	[68]
mud	–	–	gut contents	[61]
**Diet of ** ***Halicryptus spinulosus***	**higher taxa 1**	**higher taxa 2**	**source of data**	**refs**
*Halicryptus spinulosus*	Priapulida	–	gut contents	[67]
*Harmothoe sarsi*	Annelida	Polychaeta	gut contents	[67]
*Pygospio elegans*	Annelida	Polychaeta	gut contents	[67]
Naididae undet.	Annelida	Oligochaeta	gut contents	[67]
Oligochaeta undet.	Annelida	Oligochaeta	gut contents	[67]
*Monoporeia affinis*	Arthropoda (Cru.)	Amphipoda	gut contents	[67]
*Pontoporeia femorata*	Arthropoda (Cru.)	Amphipoda	gut contents	[67]
Crustacea undet.	Arthropoda	–	gut contents	[67]
Tanypodinae undet.	Arthropoda (Ins.)	Chironomidae	gut contents	[67]
Chironominae undet.	Arthropoda (Ins.)	Chironomidae	gut contents	[67]
Arthropoda undet.	–	–	gut contents	[67]
animal remains undet.	–	–	gut contents	[67]
eggs	–	–	gut contents	[67]
algal remains undet.	–	–	gut contents	[67]
detritus	–	–	gut contents	[67]

Abbreviations: Cru, Crustacea; exp, experiments; Ins, Insecta,

The recognition of genuinely generalist feeding strategies, as seen here in *Ottoia*, reveals a high level of trophic complexity and flexibility that has no equivalent in preceding eras (e.g., Ediacaran ecosystem; [70], [71]) and foreshadows modern-style ecosystems. Direct documentation of this behavior in the immediate aftermath of the Cambrian explosion indicates that the marine ecosystem had already become too complex to be understood in terms of simple linear dynamics. More likely, the ecosystem already functioned as an interactive web, with multiple interactions between animal species and the exploitation of diverse food sources. This mode of functioning, which probably set up in the Early Cambrian, is likely to have generated important feedback and accelerating effects on diversity, ecosystem stability and macroevolutionary dynamics.

### Early Onset of Parallel Trophic Pathways

Predation was undoubtedly one of the driving forces in the early diversification of metazoans and the build-up of complex animal interactions and trophic web [12], [16], [19], [29], [35], [72]. Grazing [11], [20] and suspensivory [73] were also major feeding techniques used by numerous Cambrian animals. The case of *Ottoia* highlights the role of scavenging as another key-consumption mode. We think that the rise of epibenthic communities [2] resulting from the Cambrian radiation fuelled the food web with a new pool of detrital material that became a major and abundant food source for numerous scavengers and detritivores thus promoting and boosting the detrital pathway. The input of animal-derived organic matter into the ecosystem probably deeply modified the food supply in terms of quantity, energy, chemical quality and digestibility with probable feedback effects on the evolution of digestive systems [21] and feeding modes. In common with *Ottoia*, arthropods may have acquired adaptations to exploiting this detrital food store with relatively low cost of energy expenditure. This requires testing from detailed studies on the digestive systems and appendage functionalities of Cambrian arthropods and their possible modern analogues. Parallel circuits such as the “green pathway” (through primary producers, herbivore/grazers to carnivores) and the detrital pathway that is essential in the energy flow of modern marine ecosystems [41] may have been already operating in the Cambrian adding to the trophic complexity.

## Supporting Information

Figure S1 **Sponge species that co-occur with** ***Ottoia prolifica*** **in level -120** [**2**], [42], [**52**] **of the Walcott Quarry (Burgess Shale Formation, middle Cambrian).** A, B, *Hazelia nodulifera* Walcott, ROM 40317B(1), general view and details. C, D, *Hazelia palmata* Walcott, ROM 53585, general view in polarized light and details of closely packed spicules. E, F, *Falospongia falata* Rigby, ROM 40317B(2), general view and details of skeletal tracts. G, H, *Pirania muricata* Walcott, ROM 53309, general view and details of radiating spicules. I–K, *Diagonella hindei* Walcott, ROM 61766, general view and details of the spicule network of a small and larger specimen on the same slab. L, *Eiffelia globosa* Walcott, ROM 53567, details of six-rayed spicules. msp, monaxial spicule; rsp, radial thick spicule; rtr, radial tract; tr, tract composed of numerous spicules. (Scale bar, 2 mm for *A, C, E, G, I;* 1 mm F, H, J-L; 500 µm for *B, D.*
(PDF)

Table S1 **Studied material (** ***Ottoia prolifica*** **from the middle Cambrian Burgess Shale, British Columbia, Canada).** The specimens are housed in the Royal Ontario Museum (ROM), Toronto, Canada and the Smithsonian National Museum of Natural History (originally US National Museum; USNM), Washington D.C. All the specimens have preserved digestive tracts with or without gut contents.
(XLS)
