# Supplementary material for: Gut Contents as Direct Indicators for Trophic Relationships in the Cambrian Marine Ecosystem
Source: PLoS One. 2012 Dec 26;7(12):e52200. doi: 10.1371/journal.pone.0052200 (PMC3530608; doi:10.1371/journal.pone.0052200)
Supplement: Figure S1 — Sponge species that co-occur with Ottoia prolifica in level -120 [2], [42], [52] of the Walcott Quarry (Burgess Shale Formation, middle Cambrian). A, B, Hazelia nodulifera Walcott, ROM 40317B(1), general view and details. C, D, Hazelia palmata Walcott, ROM 53585, general view in polarized light and details of closely packed spicules. E, F, Falospongia falata Rigby, ROM 40317B(2), general view and details of skeletal tracts. G, H, Pirania muricata Walcott, ROM 53309, general view and details of radiating spicules. I–K, Diagonella hindei Walcott, ROM 61766, general view and details of the spicule network of a small and larger specimen on the same slab. L, Eiffelia globosa Walcott, ROM 53567, details of six-rayed spicules. msp, monaxial spicule; rsp, radial thick spicule; rtr, radial tract; tr, tract composed of numerous spicules. (Scale bar, 2 mm for A, C, E, G, I; 1 mm F, H, J-L; 500 µm for B, D. (PDF) [file pone.0052200.s001.pdf]

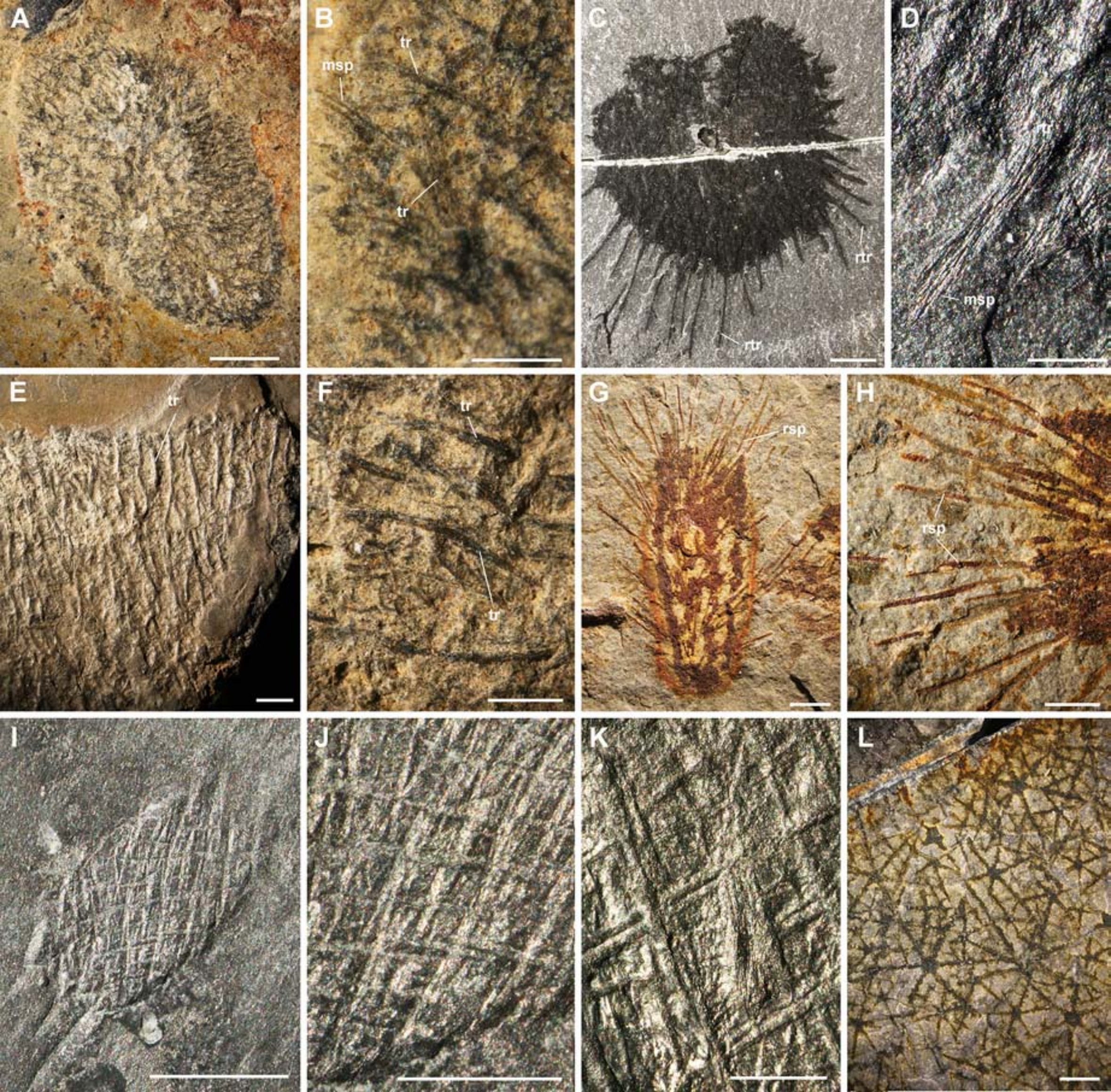
A
B
msp
tr
tr
C
rtr
rtr
D
tr
msp
E
tr
F
tr
tr
G
rsp
H
rsp
I
J
K
L

Fig. S1. Sponge species that co-occur with *Ottoia prolifica* in level -120 [2, 42, 52] of the Walcott Quarry (Burgess Shale Formation, middle Cambrian). A, B, *Hazelia nodulifera* Walcott, ROM 40317B(1), general view and details. C, D, *Hazelia palmata* Walcott, ROM 53585, general view in polarized light and details of closely packed spicules. E, F, *Falospongia falata* Rigby, ROM 40317B(2), general view and details of skeletal tracts. G, H, *Pirania muricata* Walcott, ROM 53309, general view and details of radiating spicules. I-K, *Diagonella hindei* Walcott, ROM 61766, general view and details of the spicule network of a small and larger specimen on the same slab. L, *Eiffelia globosa* Walcott, ROM 53567, details of six-rayed spicules. msp, monaxial spicule; rsp, radial thick spicule; rtr, radial tract; tr, tract composed of numerous spicules. (Scale bar, 2 mm for *A, C, E, G, I;* 1 mm F, H, J-L; 500 µm for *B, D.*

Morphology and size of sponge spicules **[57, 58]**

A-DEMOSPONGEA

Crumilospongia biporosa is sac-like, globular sponge with a well-developed canal system bearing numerous openings. The spicules are long, straight, monaxial and arranged in paralell vertical tracts (diameter of spicules (DS)= 15-20 µm).

Falospongia falata has prominent radiating or longitudinal skeletal tracts composed of numerous closely spaced parallel monaxial spicules (DS= 15-20 µm).

Hazelia conferta is a thin-walled sponge with vertically elongate monaxial spicules and radiating tufted clusters of monaxial spicules (tuft diameter DTF= ca 0.2 mm; DS= 15-20 µm).

Hazelia crateria is an elliptical broad sponge with outer part of the skeleton composed of tufts (DTF= 0.10-0.15 mm). Spicules (oxeas ; length (LS)= 0.4-0.5 mm; DS= 10-20 µm).

Hazelia delicatula is a branching tubular sponge with tufted skeleton (DTF= 10-15 µm) made of minute double-tapered oxeas (max DS= 20 µm).

Hazelia nodulifera is a frondescent to massive subhemisphaerical sponge with a skeleton composed of tracts (DTR=ca. 2 mm; DT= 0.15-0.20 mm) of dominantly monaxial subparallel spicules. Spicules with LS= 0.2 mm and DS= 15-20 µm.

Hazelia palmata is a low sponge in which the skeleton is made of irregular anastomosing radial tracts (DTR=0.2-0.3 mm) composed of subparallel oxeas (fine monaxial needle-shaped spicules; LS= 0.6 mm; DS= 20 µm).

Leptomitus lineatus is a very large double-walled tubular sponge with simple monaxial spicules arranged vertically. Outer layer with coarse spicules (LS= 25 mm).

Pirania muricata is a thick-walled cylindrical sponge with very long monaxial, thick radiating spicules (diameter > 100 µm; length 7-8 mm). Wall with tufts (DTF= 0.10-0.15 mm).

Takakkawia lineata is conical sponge with 8 twisted vertical ribbons of elongate, monaxial spicules.

B- HEXACTINELLIDA

Diagoniella hindei is an hexactinellid sponge with a simple sac-like shape and a network of spicules (stauracts) arranged diagonal to the long axis.

Protospongia hicksi is an early representative of the hexactinellid sponges. Its shape is globular to conical. The wall has a single layer of cross-shaped spicules (stauracts).

C- CALCAREA

Eiffelia globosa is globular hexactinellid sponge with a body supported by a network of six-rayed calcareous spicules of at least four different size categories.
